# Distribution and community composition of lichens on mature mangroves (*Avicennia marina* subsp. *australasica* (Walp.) J.Everett) in New Zealand

**DOI:** 10.1371/journal.pone.0180525

**Published:** 2017-06-30

**Authors:** Christy L. Reynolds, Orhan A. H. Er, Linton Winder, Dan J. Blanchon

**Affiliations:** 1 Biodiversity Management and Animal Welfare Research Group, Environmental and Animal Sciences, Unitec Institute of Technology, Auckland, New Zealand; 2 Department of Forestry and Resource Management, Toi Ohomai Institute of Technology, Rotorua, New Zealand; University of Waikato, NEW ZEALAND

## Abstract

Mangrove forests of a single trees species, *Avicennia marina* subsp. *australasica* are widespread in the upper North Island of New Zealand, but there is little available information on the diversity of epiphytes such as lichens within them. A survey of 200 trees from 20 mangrove sites recorded a total of 106 lichen species from 45 genera. Two of these species are considered to be ‘Threatened’, five ‘At Risk’ and 27 ‘Data Deficient’. Multiple regression indicated that tree diameter (DBH) and mean annual rain days positively influenced site species richness. Multidimensional scaling showed that sites from the same geographical region generally formed distinct clusters. Redundancy analysis indicated that mean annual wet days, latitude and DBH measurably influenced species composition.

## Introduction

The term “mangrove” covers a range of halophytic evergreen plants comprising over 70 species, found in 27 genera from 20 families and nine orders [1–4]. About a quarter of all mangrove species belong to the pantropical genus *Avicennia* [1]. The only mangrove species currently found in New Zealand is the Mānawa (*Avicennia marina* subsp. *australasica*), which is also found in Southeastern Australia [5]. Mangrove forests are distributed throughout the globe in 118 tropical and sub-tropical countries [6], ranging from 31°45’N in southern Japan, to 38°03’S in the North Island of New Zealand. In New Zealand, the species is naturally distributed from the top of the North Island to Kāwhia on the west coast and Ohiwa on the east coast [5].

Mangrove ecosystems occur at the convergence of terrestrial and marine communities [2], receiving saline and fresh water, sediment, and nutrient inputs from both the ocean and rivers [4]. Worldwide, mangroves are highly productive ecosystems [3, 7, 8] that provide habitat and an important source of nutrients for a variety of species [9, 10]. Mangroves are valuable nursery grounds and breeding sites for birds, fish, crustaceans, shellfish, reptiles and mammals [8]. They provide habitats for motile or visiting fauna, and support coastal fisheries through the provision of nursery areas [11]. Mangroves also filter sediment and contaminant runoff from the land into the sea, act as carbon sinks, and can store more carbon than freshwater wetlands [4, 12]. Whilst the biodiversity value of mangroves is well accepted worldwide [3, 11, 13], there is an admitted shortfall of information on the biodiversity status of New Zealand mangrove forests [14]. The State of New Zealand’s Environment Report [15] described New Zealand mangroves as being “*low in diversity*”, although 30 fish species are noted as being associated with mangroves and a wide range of native and introduced birds are known to utilize them as habitat [14]. Some terrestrial invertebrates have been recorded from mangroves in New Zealand. This includes several moth species, including *Planotortrix avicenniae*, a mite (*Acalitus avicenniae*), the lemon tree borer (*Oemona hirta*) and several ant species [14]. Despite several international studies describing epiphytic plant and/or lichen diversity on mangroves [13, 16–18], there is very little published information on epiphytes of mangroves in New Zealand. One study [19] recorded 32 lichen species from mangroves on Great Barrier Island and another [20] reported 33 species at Miranda in the Firth of Thames, but, both of these studies were small-scale and not quantitative. One threatened species, the ‘Nationally Endangered’ *Ramalina pacifica* is mainly found in mangrove forest in New Zealand [21].

Worldwide, over 90% of mangroves are found within the territory of developing countries [22] Since the 1980s at least 35% of mangrove forests have been lost, mainly due to human influences [23, 24]. The main causes of mangrove loss are considered to be mariculture, logging for timber, and removal for the establishment of agricultural systems [10, 23, 25]. In contrast to the global trend of loss, mangroves in New Zealand have been steadily spreading [5, 26, 27]. This spread is attributed to increased sedimentation caused by erosion from urbanization and agricultural development [9] This has led to public submissions for removal of mangroves [5], some of which have occurred [28].

Whilst a number of lichen species have been recorded from New Zealand mangroves [29, 30], no systematic study has been carried out on the diversity of lichens epiphytic on *Avicennia marina* subsp. *australasica*. We therefore conducted a study of the species richness, abundance and community composition of lichens in association with mangrove forest at 20 sites across its range in the upper North Island of New Zealand. We compared these assemblages with environmental and site factors in order to develop an understanding of variables that may influence their distribution.

## Methods

### Site selection

**Fig 1:**
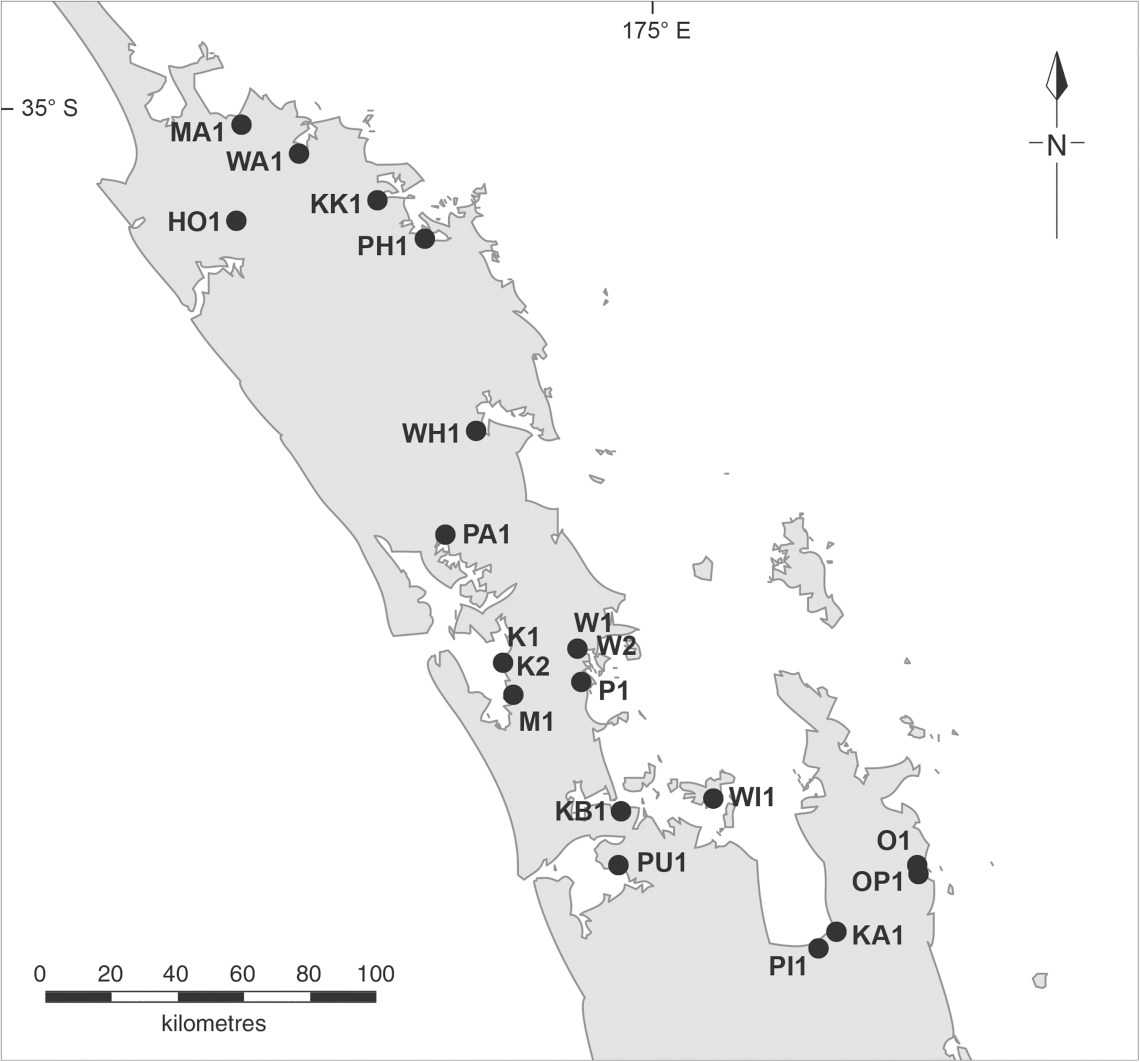
Mangrove study sites, North Island, New Zealand.

Sites were chosen from harbour areas throughout the range of *Avicennia marina* subsp. *australasica* in New Zealand (Fig 1). The sites represented four distinct areas of the upper North Island: the Far North; Coromandel Peninsula; Kaipara Harbour and mid Northland; and Auckland. The study focused on larger mature mangroves with significant trunks, as these are more likely to host epiphytes than younger saplings and shrubs with dense canopies. Sites were selected to provide representative regional spread, but, were only included if they were both accessible and contained a minimum of 40 trees (in order to provide a large enough parent population for randomised tree selection). Permission was sought from land owners to access any site.

### Lichen sampling

Ten trees at each site were randomly selected from those present and mean DBH was recorded at each site (Table 1). Lichen species observed on each individual tree sampled were recorded (from ground level to the top of the canopy), by careful visual observation to account for any vertical zonation of different species [31]. Frequency of occurrence for a given species at a site was estimated by calculating the proportion of trees that were hosts. A single tree was considered to be a host if at least a single thallus was observed. Voucher specimens of all lichen species present were collected (where possible) and accessioned into the Unitec Herbarium. Nomenclature follows the last Flora treatment and recent updates for individual genera [21, 30, 32–34].

**Table 1:** Summary of environmental and other variables.

Location	Site	Canopy Cover %	Latitude	Longitude	Min temp ^°^C	Max temp ^°^C	Mean temp °C	Mean annual rain days	Mean annual wet days	Mean annual rainfall (mm)	Mean DBH mm	Number of spp. /site
Far North	Hokianga (HO)	68	-35.272066	173.522483	11.8	19.5	15.7	198.67	163.11	1394.1	219.6	36
	Whangaroa (WA)	84	-35.084144	173.719894	11.8	19.5	15.7	182.50	132.13	1394.1	159.2	28
	Mangonui (MA)	46	-35.012327	173.560641	11.8	19.5	15.7	182.50	132.13	1394.1	216.8	29
	Kerikeri (KK)	87	-35.217416	173.985469	10.6	20.1	15.3	201.46	131.64	1709.8	231.9	33
	Paihia (PH)	82	-35.304761	174.101969	10.6	20.1	15.3	201.46	131.64	1709.8	147.7	30
Auckland	Kepa Bush (KB)	66	-36.864341	174.829175	11.3	19.0	15.2	194.17	135.71	1213.2	176.2	25
	Puhinui (PU)	59	-36.996244	174.832138	11.8	18.9	15.4	168.40	121.00	1125.2	122.6	19
	Waiheke Island (WI)	62.5	-36.824580	175.138752	11.3	18.8	15.2	194.17	135.71	1213.2	162.3	31
Kaipara/	Whangarei (WH)	100	-35.836300	174.305880	11.8	19.7	15.8	188.70	131.35	1299.9	140.6	23
Northland	Mataia (K1)	47	-36.493261	174.419052	10.2	18.8	14.5	184.81	134.94	1435.5	268.4	37
	Mataia (K2)	17	-36.490327	174.415294	10.2	18.8	14.5	184.81	134.94	1435.5	219.4	39
	Makarau (M)	69	-36.547583	174.465691	10.2	18.8	14.5	184.81	134.94	1435.5	216	38
	Warkworth (W1)	100	-36.398819	174.670513	10.2	18.8	14.5	184.81	134.94	1435.5	175.5	25
	Warkworth (W2)	94	-36.402461	174.675336	10.2	18.8	14.5	184.81	134.94	1435.5	185	19
	Puhoi (P)	9	-36.523405	174.676750	10.2	18.8	14.5	184.81	134.94	1435.5	217.6	30
	Paparoa (PA)	71	-36.114686	174.229697	10	19.1	14.5	190.96	137.39	1442.8	161.2	37
Coromandel	Kauaeranga River (KA)	72	-37.149852	175.548430	10.6	19.5	15.0	170.22	118.22	1141.4	166.4	18
Peninsula	Oturu (O)	75	-37.028222	175.833086	10.1	19.3	14.7	212.56	142.56	1839.8	103.2	26
	Orchard Point (OP)	27	-37.036219	175.842200	10.1	19.3	14.7	212.56	142.56	1839.8	112	24
	Piako Rover (PI)	77	-37.201483	174.676750	10.6	19.5	15.0	170.22	118.22	1141.4	135.1	20

### Environmental and site variables

Longitude and latitude were recorded for each of the 20 sites. Climate variables were determined from annual means (1981–2010) from the nearest weather station (taken from the NIWA CliFlo database) and comprised: mean daily minimum and maximum air temperature (^°^C), mean annual rainfall (mm), mean annual rain days (≥0.1 mm of rain), and mean annual wet days (≥1.0 mm of rain) (Table 1). The percentage mangrove canopy cover for each site was also determined using recent aerial photographs.

### Data analysis

**Fig 2:**
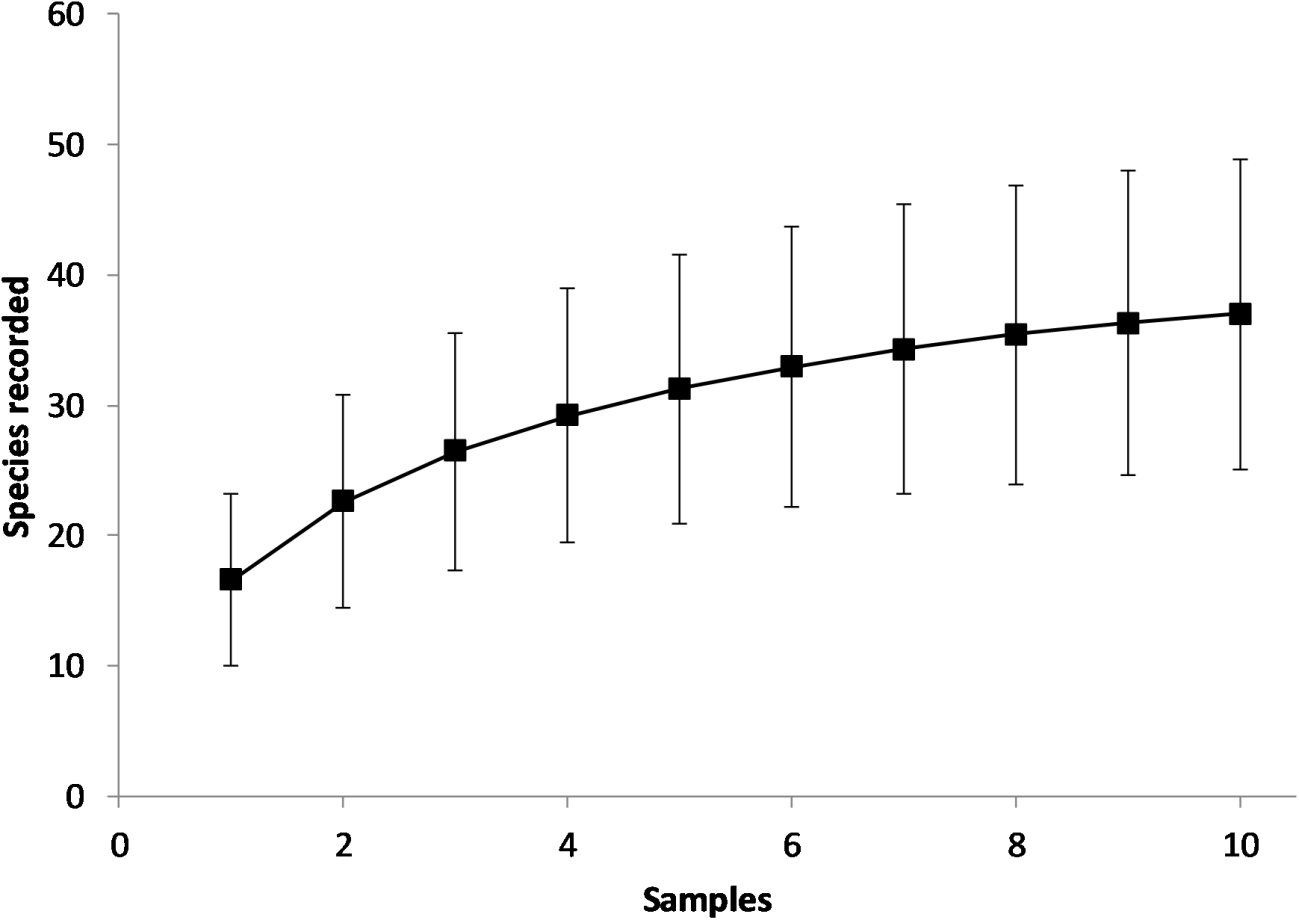
Example of a representative species accumulation curve (site K1). All other sites followed a similar pattern. Error bars represent standard deviation.

As a preliminary analysis, species accumulation curves were plotted to determine whether sampling was sufficient to adequately represent species composition. Species accumulation curves were generated using the “Sample Interpolation” method available in the package Species Diversity and Richness (version 4.1.2). This analysis indicated that ten samples were able to adequately characterise species composition (Fig 2), because the accumulation curve reached a plateau as sample size increased. Multiple regression analysis was used to evaluate the relationship between species richness (R) and environmental variables (including DBH). Automatic forward selection was done on untransformed data. Secondly, correlation coefficients were calculated for each pair of environmental variables using Pearson’s test. Regression and correlation analyses were done using Minitab (Version 17). Thirdly, multivariate analyses were conducted to explore patterns between sites, species and measured environmental variables. For these analyses, untransformed frequency of occurrence was used as an abundance measure, which ranged from 0 to 1 in intervals of 0.1 (preliminary analysis indicated that transformation had little effect on ordinations). We considered frequency of occurrence to be preferable to utilising the cover measurements which were both difficult to reliably estimate (given the spatial complexity of the host tree), and difficult to translate into values that can be used for analysis purposes [35]. Multi-Dimensional Scaling (MDS) was done to determine similarities between sites using the Bray-Curtis method (Primer, Version 6). Redundancy Analysis (RDA) was done using CANOCO Version 4.5 [36]. RDA was used to determine the relationship between the environmental variables and species composition for both sites and individual species. Use of a linear method was confirmed from a preliminary analysis. Environmental variables were selected using an iterative manual selection process in order to establish the most parsimonious model. Only variables that significantly influenced species composition were added to the model during the stepwise selection process. CANODRAW Version 4.14 was used to generate site/environment and species/environment biplots.

## Results

**Fig 3:**
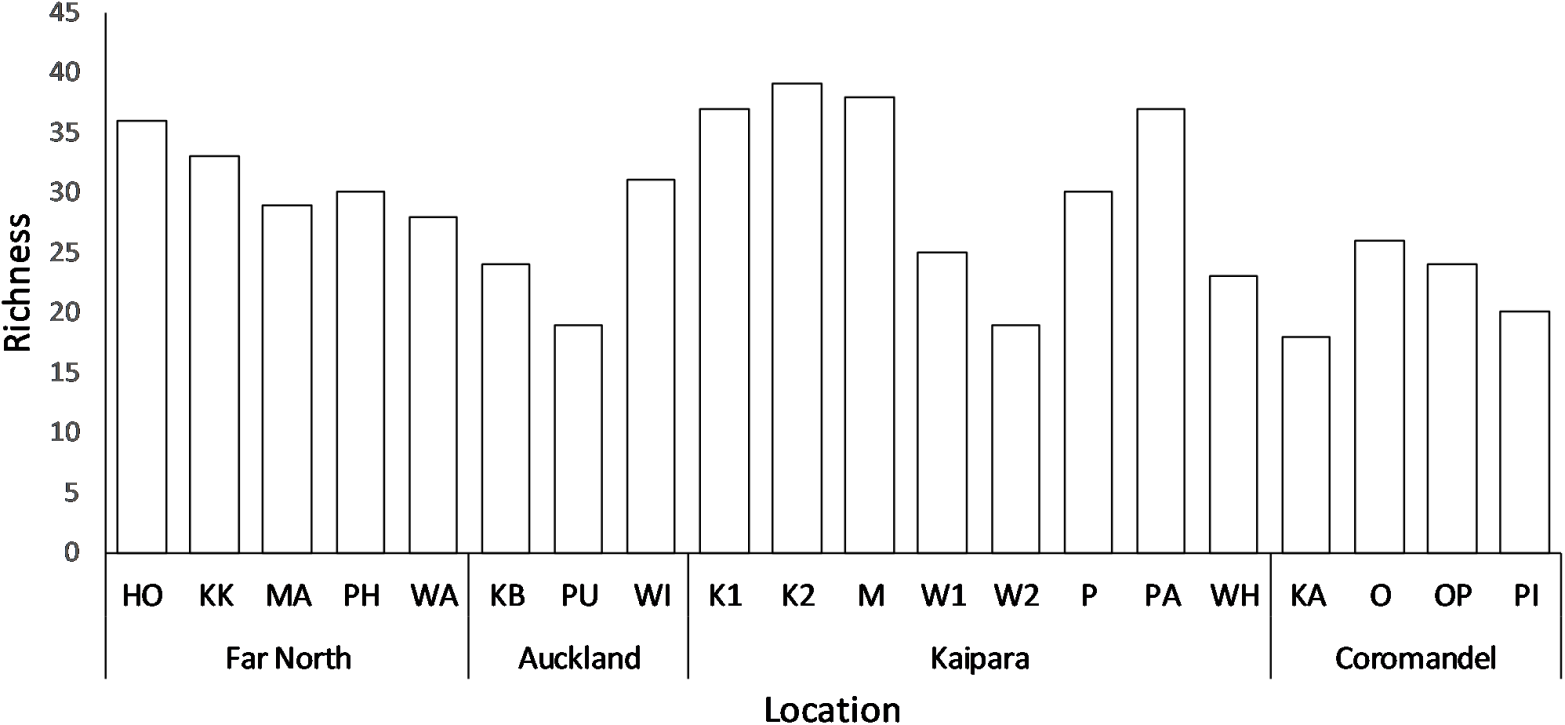
Species richness of each site surveyed within four areas of the North Island, New Zealand.

A total of 106 species of lichenized fungi within 45 genera were collected from the 200 trees sampled across the 20 sites in this study (S1 Table). The most represented family of lichens found on mangroves was the Parmeliaceae with sixteen species, followed by the Physciaceae and Lobariaceae with 15 and 13 species respectively. The most common genera were *Pseudocyphellaria*, *Pertusaria*, and *Heterodermia* with seven, *Ramalina* with six, and *Parmotrema* with five species respectively. Species richness per site ranged from 18 at the Kauaeranga River (KA) on the Coromandel Peninsula to 39 at Mataia (K2) in the Kaipara Harbour (Fig 3; S1 Table).

The most common lichen species were *Parmotrema reticulatum*, which occurred at all sites and both *Pertusaria melaleucoides* and *Parmotrema crinitum* which were recorded at 18 sites. Other common species were *Pannaria elixii*, *Physcia tribacoides*, and *Heterodermia japonica*. Of all the species identified, two were ‘Nationally Endangered’, five were ‘Naturally Uncommon’, twenty-seven were ‘Data Deficient’, sixty-nine were ‘Not Threatened’ following the designations from the most recent conservation threat classification assessment [21]. Four taxa were unable to be identified to species level, and one (*Pertusaria puffina*) has been added to the New Zealand lichen flora since publication of the threat classification for lichens [21, 37] (S1 Table). The ‘Nationally Endangered’ *Ramalina pacifica*, was found at seven sites. ‘Naturally Uncommon’ species, such as *Ramalina canariensis*, *Crocodia poculifera*, and *Pseudocyphellaria wilkinsii* were uncommon, although *P*. *wilkinsii* was found to be locally common at two of the sites were it occurred.

Some ‘Data Deficient’ lichen species were relatively common, such as *Pertusaria melaleucoides*, *Physcia tribacoides*, and *Leptogium cyanizum*. Others, such as *Lecanora argentata*, *Amandina diorista*, and *Thelotrema circumscriptum* were only rarely observed. In the genus *Leptogium* two ‘Data Deficient’ species (*L*. *cyanizum* and *L*. *phyllocarpum*) were relatively common, whilst two ‘Not Threatened’ species (*L*. *aucklandicum*, and *L*. *cyanescens*) were only rarely seen, and observed at one site each. Three ‘Data Deficient’ species in the genus *Pyrenula* were observed, with two species (*P*. *nitidula*, and *P*. *dermatodes*) present at over a quarter of all study sites (where they were commonly found). *P*. *ravenelii* was observed in abundance at two sites only.

Multiple regression analysis indicated that DBH and mean annual rain days positively influenced site species richness (R = -32.5 + 0.1069 DBH + 0.222 rain days, P_DBH_ = 0.001, P_rain days_ = 0.025). Mangrove mean DBH ranged from 103.2mm at site O (Oturu, Coromandel Peninsula) to 268.4mm at site K1 (Mataia, Kaipara Harbour). Mean annual rain days ranged from 168.4 at PU (Puhinui, Manukau Harbour) to 212.56 at O and OP (Orchard Point, Coromandel Peninsula).

**Fig 4:**
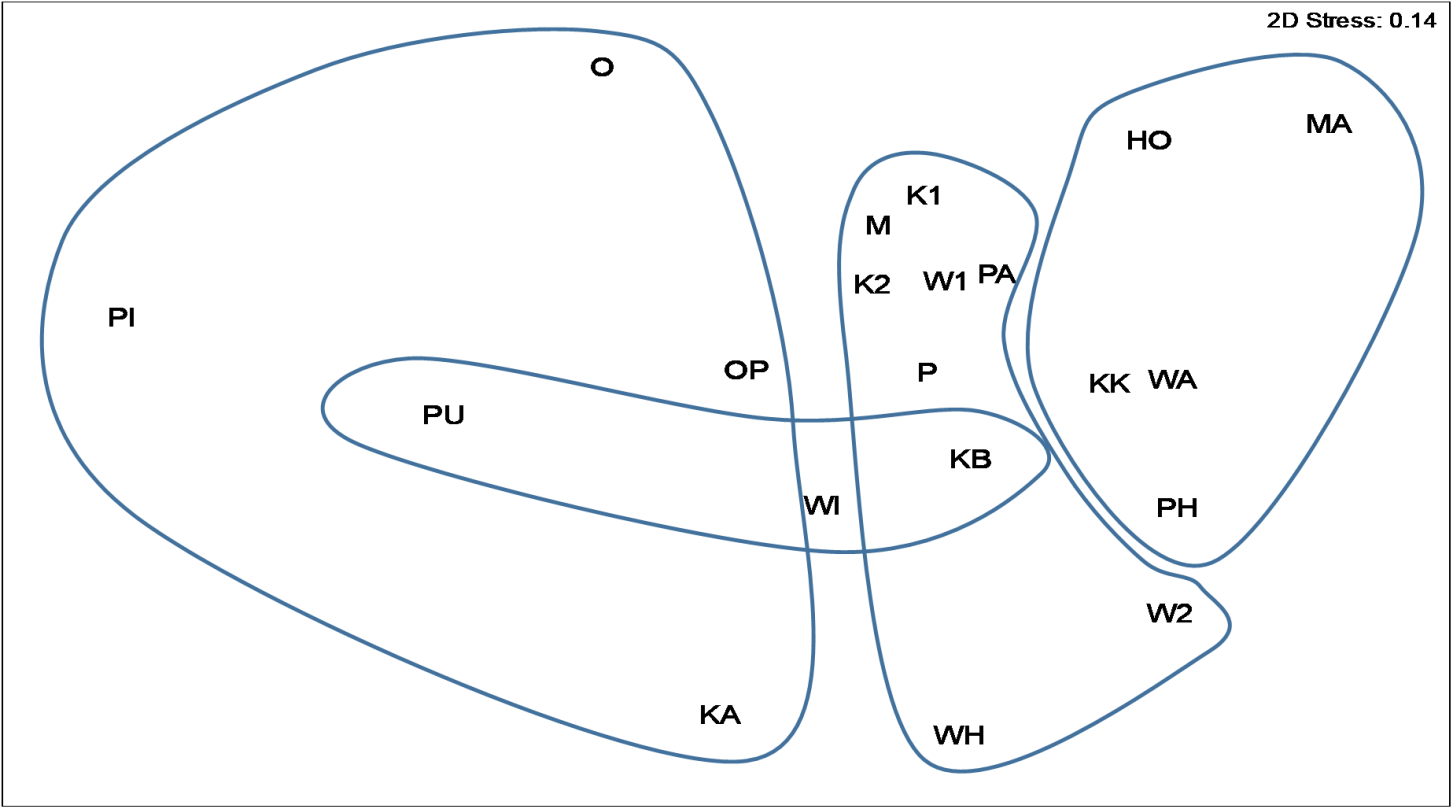
MDS plot of sites within the regions Northland (HO, KK, MA, PH, WA). **Auckland (KB, PU, WI), Kaipara (K1, K2, M, W1, W2, P, PA, WH) and Coromandel (KA O, OP, PI).** Region boundaries shown for clarity.

Environmental variables were generally not correlated (Table 2), although latitude, longitude, mean air, min. air, max. rain and wet days showed some correlation with other variables. MDS analysis indicated that there was similarity between sites from the same regional location, as clusters were generally formed (Fig 4). In particular, Kaipara Harbour/mid Northland sites showed distinct similarity and formed a recognizable cluster, with the exception of sites W2 and WH. Far North sites also formed a cluster (albeit less distinct), representing the northern-most sites studied. Coromandel sites and one of the Auckland sites (PU) also formed a diffuse cluster, representing the southern-most sites within the study. The stress value of 0.14 indicated that the analysis showed reasonable fit in two dimensions.

**Table 2:** Bivariate correlations (Pearson) between environmental variables. Significant correlations at 5% indicated by * and 1% by ** respectively.

	Lat.	Long.	Cover	DBH	Mean air	Max. air	Min. air	Max. rain	Wet days	Rain days
Lat.	-	0.90**	-0.24	-0.37	-0.56*	-0.55*	-0.42	-0.24	-0.31	-0.18
Long.		-	-0.13	-0.58**	-0.45*	-0.24	-0.44	-0.02	-0.29	0.07
Cover			-	-0.27	0.30	0.38	0.20	-0.09	-0.13	-0.03
DBH				-	-0.15	-0.20	0.09	-0.04	0.21	-0.15
Mean air					-	0.65**	0.93**	-0.28	0.00	-0.03
Max. air						-	0.32	0.25	-0.07	0.29
Min. air							-	-0.50*	0.01	-0.22
Max. rain								-	0.46*	0.81**
Wet days									-	0.68**
Rain days										-

**Fig 5:**
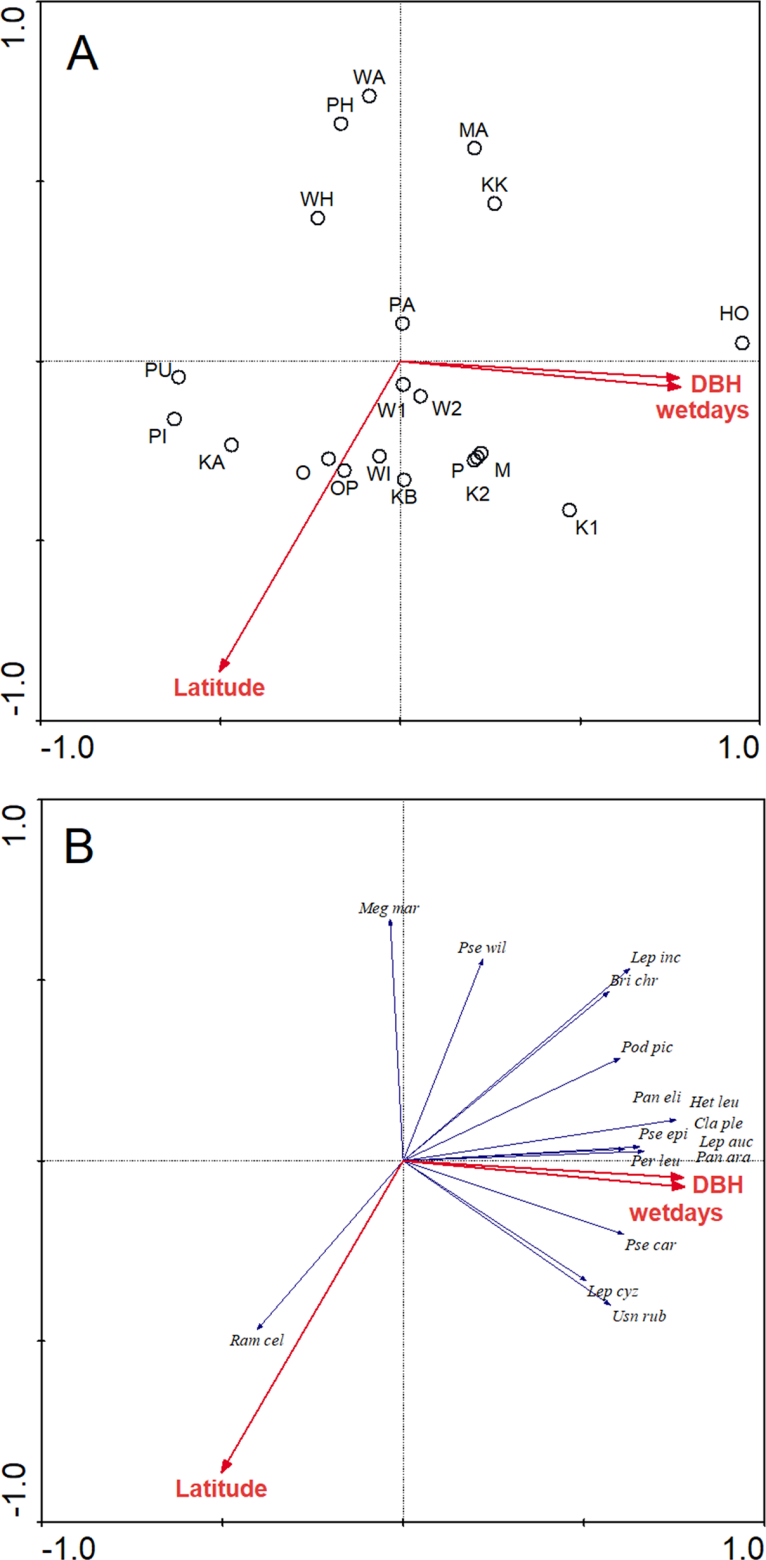
**RDA ordination plots representing relationships between influential environmental variables and sites (A) and species (B) respectively**. In the species diagram, only the sixteen species most strongly influenced by the environmental variables are displayed for clarity.

Redundancy analysis indicated that wet days (P = 0.002), latitude (P = 0.002) and DBH (P = 0.008) were good predictors of variability in species composition (Fig 5). In general, sites from the same region (and latitude) clustered together, but, not consistently. For example, distinct clustering of sites were evident (Fig 5A) for Northland sites (KK, MA, WA and PH, but, not HO), and the mid-Northland/Kaipara Harbour sites (PA, K1, K2, P, M, W1, W2, but, not WH). Two of the Coromandel sites (PI, KA) formed a cluster with one of the Auckland sites (PU) and two of the Auckland sites (KB and WI) clustered with the two eastern Coromandel sites (O and OP). The first two ordination axes accounted for 25.5% (16% and 9.5% respectively) of the observed variability in species composition (Fig 5B). Sixteen species were strongly influenced by these variables and were selected for inclusion in the species/environment plot. Fifteen species were positively related to both DBH and mean annual wet days, whilst one species (*Ramalina celastri*) was associated with increasing latitude.

## Discussion

One hundred and six species of lichens found in New Zealand mangrove forest equates to c. 6% of the known NZ lichen Flora [21], indicating that this forest type is an important habitat type for lichen diversity. Out of 38 species listed as occurring on mangroves in the most recent Flora treatment for New Zealand lichens [30], 20 were identified during this study. More species are therefore likely to be added to the checklist as more sites are investigated.

Unlike most mangrove forests, New Zealand mangrove forests are a monoculture of one tree species. Despite this, when compared with studies elsewhere in the world, the number of lichen species found in New Zealand mangroves is relatively high (as a proportion of those extant within the country). The highest recorded number of species of lichens on mangroves was recorded from a study of the southern and south-eastern coastline of Brazil, where 289 lichen taxa were reported [38]. One hundred and sixty-seven species are known from the Sundarban mangroves [39], an area of over 1 million hectares and 30–36 species of mangroves [40, 41]. An investigation of the mangroves of Trat province in southeast Thailand, which hosts 33 species of true mangroves, reported 117 species of lichens. These were found on the trunks and prop roots, and the majority of lichen species were crustose [42]. In a study of mangroves of eastern Australia, where seven species of mangroves, including *Avicennia marina*, were examined, 105 lichen species were observed [16]. However, this study focused on macrolichens, so the total number present is likely to be higher.

Other studies observed lichen richness that were generally similar to observations at individual sites in New Zealand. Twenty-nine lichen species were recorded from the Andaman Island mangroves in India [18], 21 species from mangroves on the southeast coast of India [21], seven species from Caye Caulker, Belize [43], whilst two species were recorded from Jozani-Pete mangrove creek, Zanzibar, Tanzania [44].

Patterns in lichen epiphyte diversity can be viewed as the result of interaction between environmental and habitat factors (environmental gradients, substrate tree specificity, tree size, tree age, bark characteristics) and the ability of each lichen species to disperse [45]. In our study, two variables, DBH and mean annual rain days, had a measurable effect on species richness. DBH is often used as a proxy measure of tree age [46] or overall size [47]. Variation in the diameter of the trunk of a tree affects available surface area, but also may influence the variability of the surface (bark texture and pH, water availability, presence of holes or cracks [48] and probably acts as a proxy measure of age. The positive influence of tree diameter on lichen species richness has been noted in other studies, but, not so far in studies on mangrove forests. For example, lichen species richness has been found to be greater in Mediterranean *Quercus* forest remnants where the average tree diameters were greater [49]. Similarly, a positive linear effect of trunk diameter on lichen species richness has been reported for deciduous tree stands in southern Sweden [50]. However, there is not always a clear link between tree diameter and lichen species richness. For example a study of montane rainforest in Cuba found differences in lichen species composition in relation to tree trunk diameter for one tree species, but did not observe differences for species richness, frequency or cover area [51].

Mean annual rain days (those days with 0.1mm or more of precipitation) positively influenced site species richness but maximum rainfall did not. Sites with higher mean annual number of rain days generally had more lichen species present (Table 1). The relationship between lichen growth and moisture levels is an interesting one. Lichens are poikilohydric; they cannot regulate their own water content [52]. Different lichen species obtain moisture from rain, mist, dew or high humidity [53], and most can survive periods of desiccation. As lichens dry out, the rate of photosynthesis and respiration declines until rehydration of the lichen thallus occurs [52]. Many lichens appear to function more efficiently obtaining water from dew rather than rain, and excess water can actually inhibit photosynthesis [53]. Regular small amounts of mist or rain could therefore be more optimal for the growth of most lichen species than irregular heavy rainfall events, which may explain the lack of correlation between species richness and maximum rainfall. Mean annual rain days influenced the occurrence of a number of lichen species on mangroves in Eastern Australia [16].

MDS analysis showed that sites from the same region generally formed distinct clusters (Fig 4) and the RDA identified that wet days, latitude and DBH were predictors of species composition (Fig 5). While there was a large degree of overlap in species occurences between sites (S1 Table), some lichen species were more commonly observed at certain sites, whilst some were absent. For example, *Flavoparmelia haywardiorum* was characteristic of the majority of the Kaipara Harbour sites, and was not found elsewhere. Other species that seemed to characterise the mangroves of the Kaipara harbour included *Leptogium cyanizum* and *L*. *phyllocarpum*, *Pertusaria sorodes* and *Dufourea ligulata*. This last species is an unusual occurrence, as it is usually saxicolous [30], but, it is possible that the hard bases of the trunks of mangroves are providing a similar microhabitat to that of coastal rocks. Also of note was the high diversity of species in the Lobariaceae at Kaipara sites, with seven species present. The Coromandel Peninsula sites are perhaps best characterised by what is missing. There were no *Opegrapha* species, *Ochrolechia pallescens* was absent and the Lobariaceae were also largely absent. Characteristically “northern” species of *Ramalina*, such as *R*. *australiensis* and *R*. *pacifica* [29] were also not found at these sites.

A range of possible explanatory variables for the lichen assemblages observed were examined, but, redundancy analysis revealed that only three environmental variables (DBH, latitude and mean annual wet days) were predictors of species and site composition. The causal relationships remain unknown from this study, but, these patterns are presumably driven by climatic variations which favour certain species at given geographical locations. Similarly, species assemblages were reported to change with latitude in Australia as species replaced each other through turnover [16].

As well as finding a relationship between the environmental variables DBH and mean annual rain days with species richness, our study also demonstrated that DBH measurably influenced species composition. This has been noted in other studies of epiphytic lichens. For example, a study of epiphytic lichens in the Italian alps found that after substrate tree specificity was taken into account, tree size (DBH) and age influenced lichen species dynamics [47]. They observed that tree size affected population sizes and abundance patterns, and tree age had species-specific effects as some species prefer older or younger trees. It has been suggested that the relationship between lichen species composition and trunk diameter could be due to trees with larger trunks having more suitable microclimates on the bark and a larger surface area available for colonising lichen propagules [51]. Unfortunately, tree ages for the mangrove species used in this study could not be determined by counting tree rings [54].

Whilst mean annual rain days influenced site species richness, we observed that the number of mean annual wet days influenced species composition. Moisture levels are therefore a strong driver of the species distributions that we observed. The preference of different lichen species for different moisture levels is known to depend on a range of factors. Cyanolichens generally require liquid water for photosynthesis, whereas lichens with green algal photobionts often do not [52, 53]. In addition, fine, filamentous or fruticose lichens can take up moisture from mist or humid air rapidly, but, more compact or thicker foliose lichens may not [53] and may require liquid water to rehydrate. For example, members of the Lobariaceae such as *Pseudocyphellaria* and *Sticta* are adapted to rainier climates [53, 55]. It could be expected that sites with a high number of wet days would be richer in cyanolichens and foliose lichens such as species of Lobariaceae. This is indeed the case (S1 Table) in this study, with cyanobacterial species of *Leptogium* and *Pannaria*, and species of *Crocodia*, *Pseudocyphellaria* and *Sticta* (Lobariaceae) well-represented at these sites. The most comparable study available [16] did note several cyanolichens but no species of Lobariaceae from eastern Australian mangroves. All of the sites in the Australian study had significantly lower mean annual rain days than the sites in our study.

Some individual species were strongly influenced by environmental variables. Species such as *Heterodermia leucomela*, *Leptogium aucklandicum*, *Leptogium cyanizum*, *Pannaria elixii*, *Pannaria araneosa*, *Pertusaria leucoplaca*, *Pseudocyphellaria carpoloma* and *Usnea rubicunda* were strongly positively associated with DBH and mean annual wet days. Four of these species are cyanolichens (in this case species of *Leptogium* and *Pannaria*). The presence of lichens with cyanobacterial symbionts has previously been found to correlate with tree size [49] and cyanolichens are usually associated with wetter habitats [30, 52, 53].

*Ramalina celastri* was more evident with increasing latitude, whilst *Brigantiaea chrysosticta*, *Lepraria incana*, *Megaloblastenia marginiflexa* and *Pseudocyphellaria wilkinsii* were more evident with decreasing latitude. However, it should be noted that most of these species are found throughout New Zealand [30], and most are also found on trees other than mangroves [30, 56]. Only one lichen recorded in our study, *Caloplaca mooreae* is likely to be a mangrove-specialist.

There are other possible explanations for differences in site species assemblages. Forest continuity (the continuous occupation of a site for multiple generations), can have an effect on species richness and community composition. For example, in beech forests in southern Sweden, older stands with continuous forest through time and high substrate quantity and quality were linked to higher lichen species richness [57]. Mangrove forests are highly dynamic ecosystems, and forest continuity, whether spatial or temporal is probably not the norm. Most mangrove forests have a low, dense, shrubland of seedlings and saplings, with scattered larger, older trees, often at or above the high tide level [54, 58, 59]. As mangrove forests age, they are usually characterised by scattered large trees, canopy gaps, standing dead trees and little regeneration [60]. These sites are likely to have a high lichen diversity when compared with stands of mangrove saplings, which often do not support lichens at all. In New Zealand, mangroves have been spreading rapidly in the last 80 years due to increased sediment loads in estuaries caused by the expansion of agriculture and urbanisation [5, 9, 26, 54, 59]. The distribution, abundance and size of individual mangroves has been found to vary within and between different estuaries [58], and suitable estuarine sites are not evenly distributed in the upper North Island [26], leading to some significant distributional gaps. Large scale mortality of mangrove forests can be caused by storms, tsunamis, changes in local hydrology or salinity, erosion, frost and disease [60]. The instability through time and geographical separation of these older trees, when combined with the variable ability of different lichen species to disperse over long distances, could therefore be influencing site species richness and community composition.

Another variable not investigated in our study was the possible influence of atmospheric pollutants, in particular nitrogen in the form of ammonia. Agricultural areas with high numbers of cattle can have high background levels of ammonia, and this is known to affect lichen community composition and reduce species richness at high concentrations [61–63]. Lichen species can be catagorised as nitrophytes, oligophytes or acidophytes [62, 64, 65], with acidophytes generally declining as levels of ammonium increase, and nitrophytes being favoured. Most research on the effects of ammonia on lichen diversity has been done in the Northern Hemisphere, but some of the same species are found in New Zealand. Site PI, for example, might be an example of a site influenced by atmospheric nitrogen. This site is located in the Firth of Thames at the mouth of the Piako River, which flows through an extensive flood plain area that is heavily farmed. Three species listed as being nitrophytes in Europe [63, 65] *Hyperphyscia adglutinata*, *Ramalina canariensis* and *Xanthoria parietina* were observed, and cyanolichens were absent. Cyanolichens are known to be particularly sensitive to excess environmental nitrogen [66].

Two ‘Nationally Endangered’, five ‘Naturally Uncommon’ and 27 ‘Data Deficient’ species were amongst the 106 species found on mangroves, highlighting the biodiversity importance of this habitat type. Despite mangrove forests increasing in extent in New Zealand, human activities are impacting on habitat quality and there are indications that some lichen species characteristic of mangroves and other coastal forest types may be declining. One species with its stronghold in mangrove forests, *Ramalina pacifica*, already noted as ‘Nationally Endangered’ [21], was found at less than half of the surveyed sites. Other species such as *Teloschistes flavicans*, previously found in mangrove forests and listed as ‘Declining’ [21] were not found in any of the 20 study sites. Another previously common species, *Ramalina geniculata*, known to be common in stands of *Avicennia marina* from Auckland northwards [29, 67] was only found at 12 of 20 sites, indicating a possible decline.

## Conclusion

The mean annual number of rain days and tree diameter (DBH) positively influenced lichen species richness at the 20 mangrove forest sites surveyed in this study. There was a distinct similarity in species composition between sites from the same geographical region, and this was influenced by mean annual wet days, DBH and latitude. These results indicate that regular small amounts of precipitation may be more important for lichen diversity than overall rainfall, which did not appear to influence either lichen species richness or site species composition. The high number of lichen species found, and the number of ‘Threatened’, ‘At Risk’ or ‘Data Deficient’ species indicates that these larger mangrove trees are important habitat for lichens and are in need of conservation.

## Supporting information

S1 Table Lichen species frequency of occurrence by site. (DOCX)
