# Supplementary material for: Distribution and community composition of lichens on mature mangroves (Avicennia marina subsp. australasica (Walp.) J.Everett) in New Zealand
Source: PLoS One. 2017 Jun 30;12(6):e0180525. doi: 10.1371/journal.pone.0180525 (PMC5493409; doi:10.1371/journal.pone.0180525)
Supplement: S1 Table — (DOCX) [file pone.0180525.s001.docx]

Table S1. Lichen species frequency of occurrence by site.

|  |  |  | *Far North* | | |  | *Auckland* | | | *Kaipara/ Mid Northland*  *Kaipara/ Mid Northland* | | | | | | | | *Coromandel* | | | |
| --- | --- | --- | --- | --- | --- | --- | --- | --- | --- | --- | --- | --- | --- | --- | --- | --- | --- | --- | --- | --- | --- |
| *Species* | *Abbreviation* | *HO* | *KK* | *MA* | *PH* | *WA* | *KB* | *PU* | *WI* | *K1* | *K2* | *M* | *W1* | *W2* | *P* | *PA* | *WH* | *KA* | *O* | *OP* | *PI* |
| *Amandinea diorista* var*. hypopelidna* | *Ama dio* |  |  |  | 0.1 |  |  |  |  |  |  |  |  |  |  |  |  |  |  |  |  |
| *Arthonia cinnabarina* | *Art cin* |  |  |  |  |  |  | 0.3 |  |  |  |  |  |  |  |  |  |  |  |  |  |
| *Brigantiaea chrysosticta* | *Bri chr* | 0.6 | 0.3 | 0.4 | 0.4 |  |  |  |  |  |  |  |  | 0.2 | 0.2 |  |  |  |  |  |  |
| *Caloplaca mooreae* | *Cal moo* |  | 0.1 |  |  |  |  |  | 0.4 | 0.2 | 0.3 | 0.5 | 0.1 |  | 0.1 |  | 0.1 | 0.1 |  |  | 0.1 |
| *Chrysothrix candelaris* | *Chr can* |  | 0.1 |  | 0.1 | 0.1 |  |  | 0.1 |  | 0.2 | 0.2 |  |  | 0.2 | 0.1 |  | 0.1 | 0.2 |  |  |
| *Cladonia darwinii* | *Cla dar* |  |  |  |  |  |  |  |  | 0.2 |  |  |  |  |  |  |  |  |  |  |  |
| *Cladonia pleurota* | *Cla ple* | 0.1 |  |  |  |  |  |  |  |  |  |  |  |  |  |  |  |  |  |  |  |
| *Cladonia pyxidata* | *Cla pyx* |  |  |  |  |  |  |  |  | 0.1 |  |  |  |  |  |  |  |  |  |  |  |
| *Cliostomum griffithii* | *Cli gri* |  | 0.1 |  |  |  |  |  |  |  |  |  |  |  |  |  |  |  |  |  |  |
| *Coenogonium luteum* | *Coe lut* |  |  |  |  | 0.1 |  |  |  |  |  |  |  |  |  | 0.1 |  |  |  |  |  |
| *Crocodia aurata* | *Cro aur* | 0.1 | 0.1 | 0.1 | 0.3 |  |  |  | 0.1 | 0.6 | 0.1 | 0.4 | 0.4 |  | 0.2 | 0.3 |  | 0.2 | 0.1 | 0.7 |  |
| *Crocodia poculifera* | *Cro poc* |  |  |  |  |  |  |  |  | 0.1 |  |  |  |  |  | 0.2 |  |  |  |  |  |
| *Dirinaria aegialita* | *Dir aeg* |  |  |  |  |  |  |  |  |  |  |  |  |  |  |  |  |  |  |  | 0.8 |
| *Dirinaria applanata* | *Dir app* |  |  |  |  |  | 0.1 | 0.1 | 0.2 |  |  | 0.3 |  |  | 0.2 | 0.1 |  | 0.2 |  | 0.1 | 0.3 |
| *Dirinaria picta* | *Dir pic* |  |  |  | 0.1 |  |  |  | 0.4 |  |  |  |  |  |  |  | 0.7 | 0.9 |  |  |  |
| *Dufourea ligulata* | *Xan lig* |  |  |  |  |  |  |  |  | 0.2 | 0.2 | 0.1 |  |  |  |  |  |  |  | 0.1 |  |
| *Enterographa pallidella* | *Ent pal* |  |  |  |  |  |  |  |  |  | 0.1 |  |  |  |  |  |  |  |  |  |  |
| *Flavoparmelia haywardiana* | *Fla hay* |  |  |  |  |  |  |  |  | 0.2 | 0.1 | 0.2 | 0.1 |  | 0.1 | 0.2 |  |  |  |  |  |
| *Flavoparmelia soredians* | *Fla sor* |  |  |  |  |  | 0.1 |  |  |  | 0.2 | 0.1 |  |  |  |  | 0.1 |  | 0.6 | 0.1 |  |
| *Graphis elegans* | *Gra ele* |  | 0.1 |  | 0.1 |  | 0.1 |  |  |  |  |  |  |  |  |  |  | 0.1 |  |  | 0.6 |
| *Gyalolechia flavorubescens* | *Cal fla* |  |  |  |  |  |  |  |  |  |  |  |  |  |  |  |  |  | 0.3 |  |  |
| *Heterodermia chilensis* | *Het chi* |  | 0.1 | 0.2 |  | 0.1 | 0.2 |  |  |  |  |  |  |  |  |  |  |  |  |  |  |
| *Heterodermia japonica* | *Het jap* | 1 | 0.5 | 0.2 | 0.3 | 0.3 | 0.5 |  | 0.1 |  | 0.7 |  | 0.5 | 0.3 | 0.6 | 0.9 |  | 0.1 |  | 0.2 | 0.5 |
| *Heterodermia leucomela* | *Het leu* | 0.8 |  | 0.1 |  |  |  |  |  |  | 0.2 |  |  |  |  |  |  |  |  |  |  |
| *Heterodermia microphylla* | *Het mic* |  |  |  |  |  |  |  |  |  |  |  |  | 0.1 |  |  |  |  |  |  |  |
| *Heterodermia obscurata* | *Het obs* |  |  | 0.4 |  |  |  |  |  | 0.6 |  | 0.9 |  |  |  |  |  |  |  |  |  |
| *Heterodermia spathulifera* | *Het spa* |  | 0.1 | 0.1 |  | 0.3 |  |  |  |  |  |  | 0.6 |  |  | 0.1 |  |  |  |  |  |
| *Heterodermia speciosa* | *Het spe* |  |  | 0.4 |  |  |  | 0.9 | 0.2 |  |  |  |  |  |  |  |  |  | 0.2 |  | 0.5 |
| *Hyperphyscia adglutinata* | *Hyp adg* |  |  |  |  |  |  |  |  |  |  |  |  |  |  |  |  |  |  |  | 0.3 |
| *Lecanora argentata* | *Lec arg* |  |  |  |  |  |  |  |  |  | 0.1 | 0.1 |  |  |  |  |  |  |  |  |  |
| *Lecanora dispersa* | *Lec dis* |  | 0.6 |  | 0.4 |  | 0.7 | 0.1 | 1 | 0.9 | 0.7 | 0.5 | 0.1 | 0.3 | 0.9 | 0.5 | 1 | 0.3 | 0.3 | 0.4 | 0.5 |
| *Lecanora intumescens* | *Lec int* |  |  |  |  |  |  |  |  |  |  |  |  |  |  |  | 0.1 |  |  | 0.1 |  |
| *Lepraria incana* | *Lep inc* | 0.8 | 0.5 | 0.7 | 0.1 | 0.5 |  |  |  | 0.3 |  | 0.2 | 0.3 |  | 0.1 | 0.6 | 0.1 |  |  |  |  |
| *Lepraria lobificans* | *Lep lob* |  | 0.1 | 0.2 |  |  |  |  |  | 0.4 |  |  |  |  |  | 0.1 |  |  |  | 0.1 |  |
| *Leptogium aucklandicum* | *Lep auc* | 0.2 |  |  |  |  |  |  |  |  |  |  |  |  |  |  |  |  |  |  |  |
| *Leptogium cyanescens* | *Lep cya* |  |  |  |  |  | 0.5 |  |  |  |  |  |  |  |  |  |  |  |  |  |  |
| *Leptogium cyanizum* | *Lep cyz* | 0.8 | 0.1 | 0.2 |  |  |  | 0.3 |  | 1 | 0.8 | 0.3 | 0.9 | 0.7 | 0.5 | 1 |  | 0.3 | 0.3 | 0.1 |  |
| *Leptogium phyllocarpum* | *Lep phy* |  | 0.4 |  | 0.1 |  |  |  |  | 0.4 | 0.4 |  | 0.3 | 0.2 | 0.1 |  |  |  |  |  |  |
| *Megalaria grossa* | *Meg gro* |  | 0.7 |  | 0.6 | 0.2 | 0.1 | 0.4 | 0.4 |  |  |  |  |  |  |  | 0.1 |  |  |  | 0.2 |
| *Megalaria melanotropa* | *Meg mel* |  |  |  |  |  |  |  |  |  | 0.1 |  |  |  |  |  |  |  |  |  |  |
| *Megalaria pulverea* | *Meg pul* | 0.4 | 0.3 |  |  | 0.1 | 0.1 | 0.1 | 0.5 |  |  |  |  |  |  |  |  |  |  |  |  |
| *Megaloblastina marginiflexa* | *Meg mar* | 0.2 | 0.3 |  | 0.5 | 0.4 | 0.1 |  | 0.1 |  |  |  |  |  |  | 0.2 | 0.9 |  |  |  |  |
| *Megalospora gompholoma* subsp. *gompholoma* | *Meg gom* |  |  |  |  |  |  |  |  |  |  | 0.1 |  |  |  |  |  |  |  |  |  |
| *Menegazzia aucklandica* | *Men auc* | 0.2 |  |  | 0.3 |  |  |  |  |  |  |  | 0.1 |  |  | 0.1 |  |  |  |  |  |
| *Menegazzia neozelandica* | *Men neo* |  |  |  |  | 0.1 |  |  | 0.1 |  | 0.4 | 0.4 |  |  | 0.2 | 0.1 |  |  |  | 0.1 |  |
| *Menegazzia subpertusa* | *Men sub* |  |  |  |  |  |  |  |  |  |  |  |  |  |  |  |  |  | 0.2 |  |  |
| *Normandina pulchella* | *Nor pul* |  |  |  |  | 0.1 |  |  |  |  |  | 0.1 |  |  |  | 0.2 |  |  |  |  |  |
| *Notoparmelia erumpens* | *Par eru* |  |  |  |  |  |  |  |  |  |  | 0.1 |  |  |  |  |  |  | 0.1 |  |  |
| *Ochrolechia pallescens* | *Och pal* | 0.3 | 0.4 |  | 0.7 | 0.9 | 0.3 |  | 0.3 | 0.4 | 0.1 | 0.4 | 0.4 | 1 | 0.5 | 0.8 | 1 |  |  |  |  |
| *Opegrapha agelaeoides* | *Ope age* |  |  | 0.4 |  |  | 0.4 |  | 0.1 |  | 0.2 |  |  |  |  |  | 0.2 |  |  |  |  |
| *Opegrapha atra* | *Ope atr* | 0.1 |  | 0.1 |  |  |  |  |  |  |  | 0.1 |  |  |  |  |  |  |  |  |  |
| *Opegrapha intertexta* | *Ope int* |  |  |  |  |  |  |  |  |  |  |  |  |  |  |  | 0.2 |  |  |  |  |
| *Opegrapha stellata* | *Ope ste* |  |  |  |  |  |  |  |  | 0.2 |  |  |  |  |  |  |  |  |  |  |  |
| *Pannaria allorhiza* | *Pan all* |  |  |  |  |  |  |  |  |  |  |  |  |  | 0.1 |  |  |  |  |  |  |
| *Pannaria araneosa* | *Pan ara* | 0.9 |  |  |  | 0.1 |  |  |  | 0.2 |  |  |  |  |  |  |  |  |  |  |  |
| *Pannaria crenulata* | *Pan cre* |  |  |  |  |  |  |  |  |  |  |  |  | 0.1 |  |  |  |  |  |  |  |
| *Pannaria elixii* | *Pan eli* | 0.9 | 1 | 0.8 | 0.4 | 0.9 | 1 |  | 0.2 | 1 | 0.9 | 1 | 0.5 | 0.4 | 0.6 | 1 |  |  | 0.3 | 0.2 |  |
| *Parmotrema austrocetratum* | *Par aus* |  |  |  |  |  |  |  |  |  |  |  |  |  |  |  |  | 0.1 |  |  |  |
| *Parmotrema crinitum* | *Par cri* | 0.1 | 0.3 | 0.6 | 0.3 | 0.6 | 0.3 | 0.7 | 0.8 | 0.7 | 1 | 0.7 | 0.8 | 0.2 | 0.5 | 0.7 | 0.6 | 0.4 |  | 0.9 |  |
| *Parmotrema perlatum* | *Par per* |  |  |  |  |  |  |  |  |  |  |  |  |  |  |  |  |  | 0.6 |  | 0.9 |
| *Parmotrema reticulatum* | *Par ret* | 1 | 0.9 | 0.6 | 0.3 | 0.7 | 1 | 0.9 | 0.7 | 1 | 0.7 | 1 | 1 | 0.3 | 0.8 | 0.6 | 0.3 | 0.9 | 1 | 0.9 | 0.2 |
| *Parmotrema subtinctorium* | *Par sub* |  |  |  |  |  |  |  |  |  |  |  |  |  |  |  |  | 0.1 |  |  |  |
| *Pertusaria alboatra* | *Per alb* |  |  |  |  |  |  |  |  |  | 0.5 |  |  |  |  |  |  |  |  | 0.1 |  |
| *Pertusaria leucoplaca* | *Per leu* | 0.1 |  |  |  |  |  |  |  |  |  |  |  |  |  |  |  |  |  |  |  |
| *Pertusaria melaleucoides* | *Per mel* | 0.4 | 0.5 | 0.2 | 0.6 | 0.4 | 0.8 | 0.1 | 0.7 | 0.5 | 0.2 | 0.7 | 0.5 | 0.9 | 0.8 | 0.5 | 0.4 | 0.8 |  | 0.6 |  |
| *Pertusaria psoromica* | *Per pso* |  |  | 0.1 |  |  |  |  |  |  | 0.2 |  |  |  | 0.1 |  |  |  |  |  |  |
| *Pertusaria puffina* | *Per puf* |  |  |  |  |  |  |  |  |  | 0.1 |  |  |  |  |  |  |  |  |  |  |
| *Pertusaria sorodes* | *Per sor* |  |  |  |  |  |  |  | 0.2 | 0.4 | 0.2 | 0.6 |  | 0.2 | 0.2 |  |  |  | 0.2 |  |  |
| *Pertusaria thiospoda* | *Per thi* | 0.2 |  |  |  | 0.1 | 0.2 | 0.7 | 1 | 0.2 | 0.7 | 0.2 |  | 0.3 | 0.7 | 0.1 | 0.1 | 0.9 |  |  | 0.1 |
| *Pertusaria sp. #2* | *Per sp.* |  |  |  |  |  |  |  |  |  | 0.1 |  |  |  |  |  |  |  |  |  |  |
| *Phaeographis intricans* | *Pha int* |  | 0.1 |  |  |  | 0.7 | 0.7 | 0.4 |  |  |  |  |  |  |  | 0.1 | 0.1 |  |  |  |
| *Physcia erumpens* | *Phy eru* | 0.1 |  |  |  |  |  |  |  | 0.1 |  |  |  |  |  | 0.2 |  | 0.8 |  | 0.2 |  |
| *Physcia poncinsii* | *Phy pon* |  | 0.3 | 0.1 |  |  |  | 0.7 |  | 0.4 | 0.2 | 0.5 | 0.5 |  |  |  | 0.1 |  |  | 0.1 | 0.9 |
| *Physcia tribacoides* | *Phy tri* | 0.1 |  |  | 0.2 | 0.4 | 0.5 | 0.4 |  | 0.8 | 0.6 | 0.8 | 0.6 | 0.3 | 0.6 | 0.7 | 0.2 |  | 1 | 0.2 |  |
| *Podostictina pickeringii* | *Pod pic* | 0.5 |  | 0.2 | 0.1 | 0.2 |  |  |  |  |  | 0.2 |  |  | 0.1 |  |  |  | 0.1 |  |  |
| *Porina exocha* | *Por exo* |  |  | 0.1 |  |  |  |  |  |  | 0.1 |  |  |  |  |  |  |  |  |  |  |
| *Pseudocyphellaria crocata* | *Pse cro* | 0.3 |  | 0.1 |  |  |  |  | 0.1 | 0.9 |  |  |  |  |  | 0.1 |  |  |  |  |  |
| *Pseudocyphellaria carpoloma* | *Pse car* | 0.7 |  |  | 0.1 | 0.1 |  |  |  | 0.5 |  | 0.7 |  |  | 0.8 | 0.4 |  |  |  |  |  |
| *Pseudocyphellaria coriacea* | *Pse cor* | 0.3 |  |  |  |  |  |  |  |  |  |  | 0.1 |  |  |  |  |  |  |  |  |
| *Pseudocyphellaria episticta* | *Pse epi* | 0.1 |  |  |  |  |  |  |  |  |  |  |  |  |  |  |  |  |  |  |  |
| *Pseudocyphellaria c.f. pubescens* | *Pse c.f* |  |  |  |  |  |  |  |  |  |  |  |  | 0.1 |  |  |  |  |  |  |  |
| *Pseudocyphellaria wilkinsii* | *Pse wil* |  | 0.5 | 0.7 |  | 0.3 |  |  |  | 0.1 | 0.1 | 0.1 |  |  |  |  |  | 0.1 |  |  |  |
| *Punctelia borreri* | *Pun bor* |  |  |  |  |  |  | 0.2 |  |  |  |  |  |  |  |  |  |  | 0.1 |  | 0.1 |
| *Pyrenula dermatodes* | *Pyr der* |  | 0.1 |  | 0.2 |  |  | 0.3 | 0.2 |  |  |  |  |  |  | 0.2 |  |  |  |  | 0.1 |
| *Pyrenula nitidula* | *Pyr nit* | 0.1 |  |  | 0.1 |  | 0.3 |  | 0.4 |  | 0.2 |  |  |  |  | 0.2 | 0.3 |  |  |  |  |
| *Pyrenula ravenelii* | *Pyr rav* |  |  | 0.6 |  |  |  |  |  |  |  |  |  |  |  | 0.5 |  |  |  |  |  |
| *Pyrenula c.f sexluminata* | *Pyr c.f* |  |  | 0.1 |  |  |  |  |  |  |  |  |  |  |  |  |  |  |  |  |  |
| *Pyrenula* sp. #1 | *Pyr sp.* |  |  |  |  |  |  |  |  |  |  |  | 0.1 | 0.3 | 0.1 |  |  |  |  |  |  |
| *Pyrenula* sp. #2 | *Pyr sp.* |  |  | 0.1 |  |  |  |  |  |  |  |  |  |  |  |  |  |  |  |  |  |
| *Ramalina australiensis* | *Ram aus* | 0.1 |  | 0.2 | 0.2 | 0.4 |  |  | 0.5 | 1 | 0.1 | 0.1 |  |  |  | 0.1 |  |  |  |  |  |
| *Ramalina canariensis* | *Ram can* |  |  |  |  |  |  |  |  | 0.1 |  | 0.1 |  |  |  | 0.1 |  |  |  |  | 0.2 |
| *Ramalina celastri* | *Ram cel* | 0.1 |  | 0.1 |  | 0.2 | 0.1 | 1 | 0.7 | 0.5 | 0.4 | 0.7 | 0.1 |  | 0.3 | 0.5 | 0.2 |  | 1 | 0.8 | 1 |
| *Ramalina geniculata* | *Ram gen* | 0.4 | 0.2 |  | 0.1 | 0.1 |  |  | 0.3 | 0.2 |  | 0.6 | 0.7 |  | 0.4 | 0.1 |  |  | 0.9 | 0.2 |  |
| *Ramalina pacifica* | *Ram pac* | 0.1 | 0.4 |  | 0.2 |  |  |  | 0.7 | 0.8 | 0.1 | 0.2 |  |  |  |  |  |  |  |  |  |
| *Ramalina peruviana* | *Ram per* | 0.1 | 0.3 |  | 0.2 |  |  |  |  | 0.1 | 0.3 | 0.3 | 0.4 |  | 0.2 | 0.9 | 0.1 |  | 0.4 | 0.1 | 1 |
| *Sticta squamata* | *Sti squ* | 0.3 | 0.4 |  | 0.1 | 0.2 | 0.3 |  | 0.3 | 0.2 |  | 0.9 |  |  | 0.3 | 0.4 |  |  |  |  |  |
| *Teloschistes chrysophthalmus* | *Tel chr* |  |  |  |  |  |  |  |  |  |  |  |  |  |  |  | 0.1 |  | 0.2 | 0.2 | 0.2 |
| *Thalloloma subvelata* | *Tha sub* | 0.1 | 0.1 |  | 0.6 | 0.3 |  | 0.2 | 0.7 |  | 0.2 | 0.1 | 0.6 | 0.5 |  |  |  |  | 0.1 | 0.1 |  |
| *Thelotrema circumscriptum* | *The cir* |  |  |  |  |  |  |  |  |  |  |  |  |  |  |  |  |  | 0.2 |  |  |
| *Thelotrema lepadinum* | *The lep* |  | 0.1 |  |  |  |  |  |  |  |  |  |  |  |  |  |  |  |  |  |  |
| *Usnea angulata* | *Usn ang* |  |  |  |  |  |  |  |  |  |  |  |  |  |  |  |  |  | 0.3 |  |  |
| *Usnea ciliifera* | *Usn cil* |  |  |  |  |  |  |  |  |  |  |  |  |  |  | 0.1 |  |  | 0.2 |  |  |
| *Usnea cornuta* | *Usn cor* |  |  |  |  |  |  |  |  |  |  |  |  |  |  |  |  |  | 0.1 |  |  |
| *Usnea rubicunda* | *Usn rub* | 1 | 0.3 | 0.2 | 0.3 | 0.3 | 0.6 |  | 0.6 | 1 | 0.4 | 0.9 | 0.7 | 0.1 | 0.6 | 1 |  |  | 1 | 0.9 |  |
| *Xanthoria parietina* | *Xan par* |  |  |  |  |  |  | 0.8 |  | 0.1 | 0.2 |  | 0.1 |  |  |  | 0.1 |  |  |  | 0.9 |
| Unknown 1 | unknown |  |  |  |  |  | 0.1 |  |  |  |  |  |  |  |  |  |  |  |  |  |  |
